# Security Control of Cyber–Physical Systems under Cyber Attacks: A Survey

**DOI:** 10.3390/s24123815

**Published:** 2024-06-13

**Authors:** Wei Xing, Jun Shen

**Affiliations:** College of Automation Engineering, Nanjing University of Aeronautics and Astronautics, Nanjing 211106, China; xingwei@nuaa.edu.cn

**Keywords:** cyber–physical system, cyber attack, security control

## Abstract

Cyber–physical systems (CPSs), which combine computer science, control systems, and physical elements, have become essential in modern industrial and societal contexts. However, their extensive integration presents increasing security challenges, particularly due to recurring cyber attacks. Therefore, it is crucial to explore CPS security control. In this review, we systematically examine the prevalent cyber attacks affecting CPSs, such as denial of service, false data injection, and replay attacks, explaining their impacts on CPSs’ operation and integrity, as well as summarizing classic attack detection methods. Regarding CPSs’ security control approaches, we comprehensively outline protective strategies and technologies, including event-triggered control, switching control, predictive control, and optimal control. These approaches aim to effectively counter various cyber threats and strengthen CPSs’ security and resilience. Lastly, we anticipate future advancements in CPS security control, envisioning strategies to address emerging cyber risks and innovations in intelligent security control techniques.

## 1. Introduction

**Figure 1:**
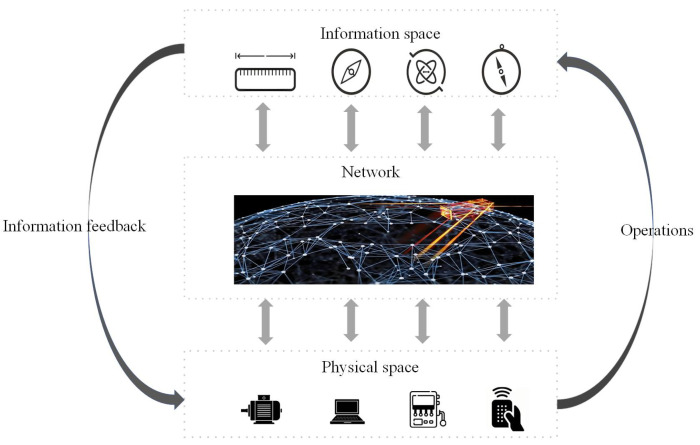
Structure of a CPS.

Amidst the continuous advancement of computer technology, network communication, and automation control, various domains are progressively integrating novel technologies and converging with physical entities [1]. For instance, distributed and embedded computing systems monitor physical processes, while, reciprocally, physical processes influence computational and communicative outcomes through feedback loops. Against this backdrop, cyber-physical systems (CPSs), embodying an amalgamation of information systems and physical systems, have come to the fore. From their conception, CPSs have garnered widespread attention from both academia and industry. CPSs incorporate functionalities such as communication, computation, and remote collaborative control [2,3,4]. Their applications hold significant value across various domains including healthcare services, intelligent transportation, smart grids, aerospace, and modern agriculture [5,6,7]. The widespread application of CPSs in practical engineering facilitates the realization of more flexible and efficient operational demands for large-scale industrial systems, thereby enhancing market competitiveness [8,9,10]. Consequently, the theoretical research and practical applications of CPSs hold profound significance, greatly advancing the global development of informatization and intelligence [11,12]. A CPS leverages advanced sensing, computing, communication, and control technologies to achieve a close integration between physical space and information space [13], see Figure 1. In this context, the physical space typically comprises the constituent elements of actual physical systems, such as sensors, actuators, and other devices [14,15]. These components facilitate the perception of the physical system’s state and enable precise control based on decision commands received from the information space. The information space generally encompasses data processing components, which conduct a computational analysis based on information gathered from the physical space. Subsequently, they provide corresponding decisions. CPSs emphasize the deep integration of physical space and information space, rendering originally closed physical devices more open. While this brings technological advantages, it also leads to an escalating security risk for the CPS.

**Figure 2:**
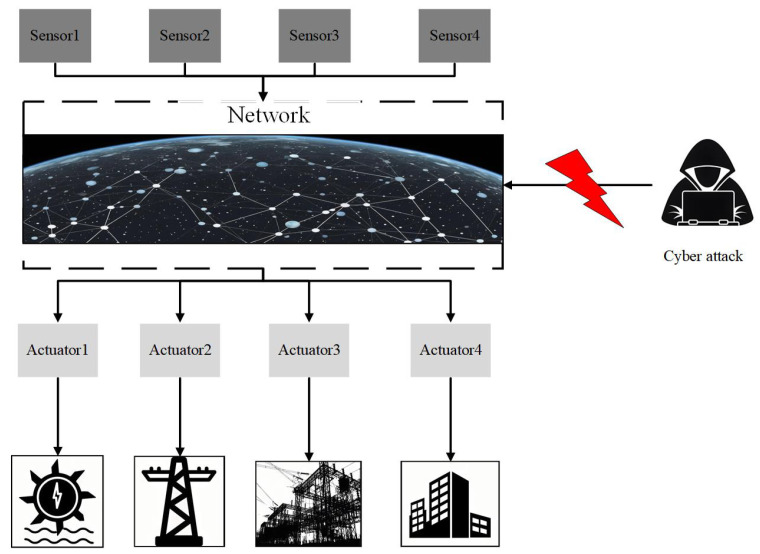
A CPS under cyber attack.

Network attacks represent an ineliminable security vulnerability that evolves alongside the advancement of network communication. Against this developmental backdrop, various forms of network attacks have emerged, including denial of service (DoS) attacks [16], false data injection (FDI) attacks [17], replay attacks [18], flooding attacks [19], masquerade attacks [20], wormhole attacks [21], and others. As shown in Figure 2, a cyber attack may attack the uplink or downlink. In recent years, major security incidents occurring worldwide have elevated the security concerns surrounding CPSs to unprecedented levels. For example, in 2003, the SQL Slammer worm virus attacked the Davis–Besse nuclear power plant in the United States. This attack disrupted the normal functioning of the nuclear power plant’s security monitoring system for an extended period by reducing network traffic [22]. In 2008, the subway system of a city in Poland fell victim to a cyber intrusion. Attackers breached the transportation control systems, enabling them to manipulate track switches directly using a television remote control, resulting in the derailment of four train cars [23]. The continuously emerging security issues posed by CPSs not only greatly impact societal prosperity and stability but also pose a potential threat to human life. Therefore, ensuring the security of CPSs is of the utmost urgency.

With the continuous emergence of security issues, both societal economic development and individuals’ livelihoods may face crises that could erupt at any moment. Moreover, the security concerns surrounding CPSs are notably intricate, encompassing not only control issues pertaining to physical processes but also technical challenges in the realms of computing and communication [24]. The growing interest and active research in this area highlight the urgency and relevance of these studies. Dibaji et al. present a structured exploration of CPS security in their study, categorizing security methods into prevention, resilience, and detection, while also introducing a unified threat assessment metric [2]. This framework aids in systematically tackling security issues by delineating specific focus areas that are essential for enhancing CPS security. In their comprehensive analysis, Wang et al. delve into recent advancements in CPS security by categorizing findings across various topics and evaluating trends in diverse dimensions and applications. Their extensive statistical analysis provides a broad perspective of the evolving research landscape, highlighting established strategies and pinpointing areas ripe for further investigation [25]. Focusing on a niche sector, Amin et al. explore cyber–physical threats within smart grids, discussing vulnerabilities and formulating mitigation strategies for power electronics systems integrated with renewable energy sources. Their targeted approach provides crucial insights into the unique security challenges of smart grids, proposing specific strategies to enhance resilience and prevent disruptions in these critical power systems [26]. Compared with the above surveys, this paper elucidates several typical attack methods and their impacts on CPSs, along with solutions encompassing state estimation, attack detection, and security control. This paper aims to systematically review security control strategies for CPSs facing cyber threats, evaluate detection methods, and explore the integration of emerging technologies into CPS security.

**Figure 3:**
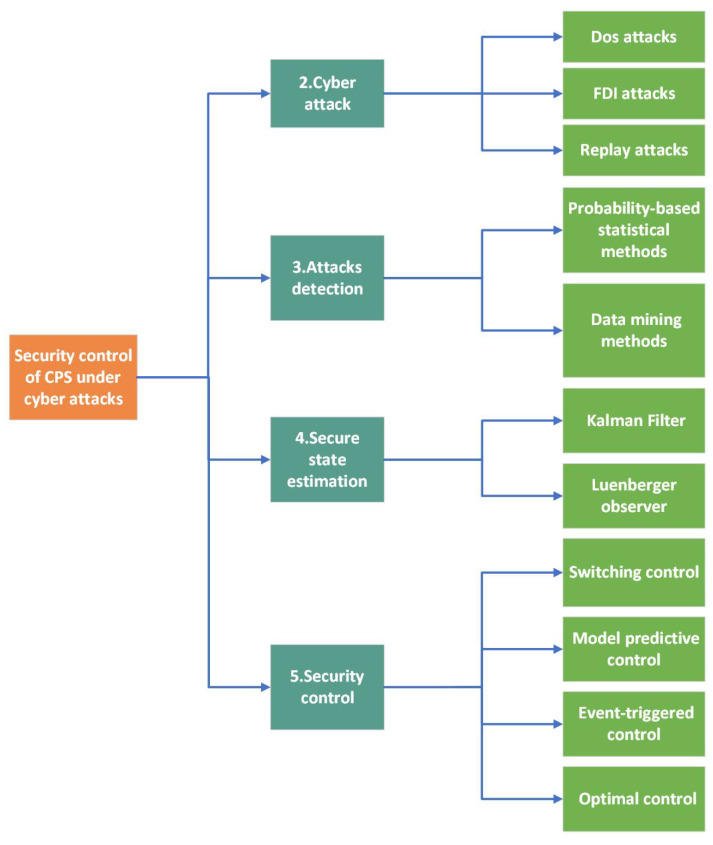
Main content framework.

The subsequent sections of this article are organized as follows: Section 2 introduces the primary cyber attacks and their impacts on CPSs, Section 3 lists classical methods of attack detection, Section 4 summarizes approaches to securinf state control, Section 5 discusses the security control methods currently in widespread use, Section 6 offers a future outlook, and Section 7 concludes the paper. Figure 3 provides a schematic diagram of the main content framework.

## 2. Cyber Attacks

Cyber attacks are differentiated by the attacker’s knowledge of the target and their impact on information layers, categorizing them into active and passive types. Active attacks disrupt system control and operations through vulnerabilities or protocol manipulation, often requiring minimal expertise and resulting in numerous incidents, including prevalent DoS and distributed DoS attacks that flood networks, slowing or crashing communications [27]. Passive attacks, on the other hand, involve secretly monitoring data without altering system information, leaving few traces and making detection challenging. Examples include replay and FDI attacks.

### 2.1. Denial of Service Attacks

DoS attack is a type of cyber attack aimed at making a target system unable to provide regular services, thereby denying legitimate users access to those services. Attackers achieve this by flooding the target system with an overwhelming amount of requests or by consuming system resources, causing it to be unable to handle legitimate user requests and thus rendering the system unavailable.

For CPSs, a DoS attack can have severe consequences. CPSs rely on real-time data collection, transmission, and processing to ensure the effective control and monitoring of physical processes. If a CPS is subjected to a DoS attack, it may result in the system being unable to respond promptly to changes in the physical environment, leading to control failures and data loss. The two most common types of DoS attack are as follows:

#### 2.1.1. Stochastic Denial of Service Attacks

In the current research, stochastic attacks are commonly modeled using either Bernoulli processes [16] or Markov processes [28], depending on the characteristics and assumptions of the attack pattern. The Bernoulli process is utilized when the occurrences of DoS attacks are assumed to be independent and identically distributed over time, with each attack occurrence being a binary event (either happening or not happening), which means $\mathrm{Prob}\left\{S(k)=1\right\}=\alpha ,\mathrm{Prob}\left\{S(k)=0\right\}=1-\alpha$. On the other hand, Markov processes model attack patterns where the current state depends on the previous state, thus allowing for memory in the attack sequence.

The influence of a DoS attack can be mathematically represented as follows:

(1) $$\begin{array}{c} \left.S\left(k\right)=\left\{\begin{array}{c} 1,\mathrm{if}\;\mathrm{DoS}\;\mathrm{attack}\;\mathrm{occurs}\;\mathrm{at}\;\mathrm{time}\;k,\\ 0,\mathrm{if}\;\mathrm{no}\;\mathrm{DoS}\;\mathrm{attack}\;\mathrm{occurs}\;\mathrm{at}\;\mathrm{time}\;k, \end{array}\right.\right. \end{array}$$

where S(k) indicates the presence of a DoS attack. When S(k)=1, the system experiences information loss as the attack disrupts the normal operation. When S(k)=0, the system operates normally, and data transmission continues without interference.

Consider a cyber–physical system modeled by the following dynamics:

(2) $$\begin{array}{c} \left\{\begin{array}{c} x(k+1)=Ax(k)+Bu(k),\\ z(k)=Cx(k), \end{array}\right. \end{array}$$

where x(k) represents the state vector of the system, u(k) is the control input, and z(k) is the output vector. The matrices A,B, and *C* define the system dynamics.

During a DoS attack, the control input u(k) is effectively nullified, which can be modeled as follows:

(3) $$\begin{array}{c} \left\{\begin{array}{c} x(k+1)=Ax(k)+(1-S(k))Bu(k),\\ z(k)=Cx(k), \end{array}\right. \end{array}$$

where (1−S(k)) acts as an attack vector. When S(k)=1 (attack occurs), the term (1−S(k))Bu(k) becomes zero, indicating that the control input Bu(k) is entirely blocked or disrupted by the attack. This leads to the system evolving without the influence of the control input, which can drastically affect the system’s performance and stability. Without the control input during an attack, the system may not respond appropriately to external changes or internal demands, possibly drifting from its desired state or becoming unstable. This explanation helps to clarify the severe consequences of DoS attacks on a CPS and underscores the importance of robust control strategies that can mitigate such disruptions.

#### 2.1.2. Periodic Denial of Service Attacks

The primary parameters of periodic attacks typically include the attack period, which defines the time interval between consecutive attacks, and the attack duration, indicating how long each attack lasts [29]. The periodic attack signal can be represented as follows:

(4) $$\begin{array}{c} \left.S\left(k\right)=\left\{\begin{array}{c} 1,nT\leq k<nT+T_{off},\\ 0,nT+T_{off}\leq k<\left(n+1\right)T, \end{array}\right.\right. \end{array}$$

where *n* is an integer representing the index of the current period, making n∈N. *T* signifies the total period duration, which is the complete cycle time of one full period of the attack pattern, including both the active and inactive phases of the attack. $T_{off}$ is the duration of the attack within each period, representing the time for which the attack is active (denoted by S(k)=1). nT represents the start time of the *n*-th attack period, and $nT+T_{off}$ marks the end time of the active attack phase within that period.

### 2.2. False Data Injection Attacks

FDI attacks are a form of attack where falsified information is injected into control systems by tampering with sensor data. By injecting false data into the control system, attackers can mislead the system, causing it to perceive normal environmental conditions, leading to erroneous control decisions. This can result in incorrect responses to the environment, thereby impacting the stability and performance of the system.

Consider a simple temperature control system for a chemical reactor, where the control system regulates the reactor temperature by adjusting the cooling fluid flow based on sensor readings. The system dynamics can be described as system (2), where x(k) represents the current temperature of the reactor and concentration of reactants, u(k) is the rate of cooling fluid flow, and z(k) is the temperature readings from the reactor, which are used to monitor and adjust the control actions.

#### 2.2.1. False Data Injection Attacks on Sensors

Specifically, when the target of FDI attacks is a sensor, the system (2) can be represented as:

(5) $$\begin{array}{c} \left\{\begin{array}{c} x(k+1)=Ax(k)+Bu(k),\\ z(k)=Cx(k)+\alpha (k)f(k), \end{array}\right. \end{array}$$

where α(k) represents the FDI attack signal, α(k)=1 indicates that the attacker has successfully injected false information into the system, and α(k)=0 denotes failure, with f(k) representing the false data. If the control system mistakenly believes the temperature is lower than it actually is due to the false sensor data, it may reduce the coolant flow unnecessarily. This can lead to overheating, risking safety and potentially causing a system shutdown or damage to the reactor. Incorrect sensor readings may lead to inappropriate control actions being taken, potentially causing instability or malfunction in the system [30].

#### 2.2.2. False Data Injection Attacks on Actuators

When attacking actuators, the system (2) can be modeled as follows:

(6) $$\begin{array}{c} \left\{\begin{array}{c} x(k+1)=Ax(k)+B(u(k)+\alpha (k)f(k)),\\ z(k)=Cx(k), \end{array}\right. \end{array}$$

Here, f(k) (if f(k)>0) falsely increases the perceived need for a coolant regardless of the actual temperature. The actuators might open the coolant valves more than necessary, leading to overcooling. This inappropriate response could result in suboptimal reaction conditions, increased energy consumption, and possibly disruptions to the chemical process. If the attack causes the actuators to perform anomalous operations, it may result in damage or the malfunctioning of the system’s physical components. This could lead to system downtime or reduced reliability, thereby impacting the normal operation of the CPS [31].

#### 2.2.3. False Data Injection Attacks on a System

Additionally, when FDI attacks the system directly, the system (2) takes on the following form:

(7) $$\begin{array}{c} \left\{\begin{array}{c} x(k+1)=Ax(k)+Bu(k)+\alpha (k)f(k),\\ z(k)=Cx(k), \end{array}\right. \end{array}$$

The falsified data f(k) directly add a misleading temperature increment or decrement to the system state. This direct manipulation can cause the controller to oscillate between overcooling and overheating, leading to instability in the reactor’s temperature. This oscillation could wear out mechanical components faster, lead to inefficient chemical reactions, or cause safety hazards due to temperature extremes. When an FDI directly attacks the control system, it leads to changes in the system model. These changes in the system model may have negative impacts on the control process, including a decreased control performance, an increased control deviation, controller failure, and system instability.

### 2.3. Replay Attacks

A replay attack is a common type of network security attack. Its fundamental principle involves an attacker intercepting previous communication traffic without authorization and then replaying it to the target system to deceive, impersonate, or replicate previous communication actions. The danger of replay attacks lies in their ability to bypass conventional authentication and access control mechanisms, allowing the unauthorized execution of operations using previously captured communication traffic. Additionally, because replay attacks do not alter data content, they are often difficult to detect and defend against.

Consider a specific example of a replay attack on an automated warehouse system (2), where robots are tasked with moving inventory based on control commands, and sensors are used to track the position and status of each robot. In the automated warehouse system, x(k) represents the positions, u(k) refers to the commands sent to robots, and z(k) is the robots’ positions.

#### 2.3.1. Replay Control Commands

Attackers might intercept legitimate control commands from previous communications and replay them at inopportune times to deceive the control system into executing unnecessary or malicious operations. The system dynamics in the face of such an attack can be modeled as follows:

(8) $$\begin{array}{c} \left\{\begin{array}{c} x\left(k+1\right)=Ax\left(k\right)+B(\left(1-\beta \left(k\right)\right)u\left(k\right)+\beta \left(k\right)u^{r}\left(k\right)),\\ z(k)=Cx(k), \end{array}\right. \end{array}$$

where β(k) denotes the replay attack signal, with β(k)=1 indicating the successful injection of historical control commands by the attacker, while β(k)=0 denotes normal system operation, with $u^{r}\left(k\right)$ representing the historical control commands. For example, if attackers replay a command that directs a robot to move inventory during a period in which such a move disrupts the logistics flow or coincides with an unauthorized withdrawal, it could result in misplaced inventory or even theft.

#### 2.3.2. Replay Sensor Data

In another form of replay attack, attackers might capture and save previous sensor data, then transmit them to the system as if they were current data at an inappropriate time. The system representation under such an attack is as follows:

(9) $$\begin{array}{c} \left\{\begin{array}{c} x(k+1)=Ax(k)+Bu(k),\\ z\left(k\right)=\left(1-\beta \left(k\right)\right)Cx\left(k\right)+\beta \left(k\right)z^{r}\left(k\right), \end{array}\right. \end{array}$$

where $z^{r}\left(k\right)$ denotes the historical sensor data. This can lead to chaos in the operational logistics of the warehouse. For instance, if the system believes that a robot is in location *P* based on replayed data when it is actually in location *Q*, any command issued based on this false information could lead to collisions, incorrect inventory placement, or delays in operation.

## 3. Attack Detection

Attack detection can help to identify potential threats early on and promptly take corresponding defensive measures to ensure the normal operation of the system. Additionally, attack detection assists in collecting attack data and behavior patterns, providing valuable information for further security analyses and defense. Therefore, attack detection in CPS is crucial for maintaining system security, ensuring data integrity and availability, and enhancing system resilience.

### 3.1. Probability-Based Statistical Methods for Attack Detection

Probability-based statistical methods involve designing a test statistic calculated from residuals for parameter testing. When an attack occurs, the statistical data of the residuals will differ from the statistical data during normal mode operation. A common detector is the chi-square detection:

(10) $$\begin{array}{c} \chi^{2}=\sum \frac{{\left(O_{i}-E_{i}\right)}^{2}}{E_{i}}, \end{array}$$

where $\chi^{2}$ represents the chi-square statistic, $O_{i}$ denotes the observed value and $E_{i}$ represents the expected value. In chi-square detection, the chi-square statistic is calculated by summing the squared differences between observed and expected values, divided by the expected values. The decision rule for determining the presence of anomalies or attacks based on the chi-square statistic can be expressed as follows:

(11) $$\begin{array}{c} \mathrm{If}\;\chi^{2}>\tau ,\;\mathrm{then}\;\mathrm{an}\;\mathrm{anomaly}\;\mathrm{or}\;\mathrm{attack}\;\mathrm{is}\;\mathrm{indicated}, \end{array}$$

where τ is a predefined threshold value, determined based on the desired sensitivity and specificity of the detection system. In Reference [32], a novel approach to integrity and continuity risk assessment for fault detection and exclusion using receiver autonomous integrity monitoring (RAIM) was provided. These methods were developed specifically for solution separation and Chi-squared RAIM. In Reference [33], using network traffic characteristics, flooding attacks were identified by statistically analyzing their chi-square, and anomalies were effectively detected when the calculated value fell below a set threshold.

Another method is covariance detection. For example, the method based on mean and standard deviation can be used to detect anomalies. Assuming μ and σ are the mean and standard deviation of covariance, respectively, anomalies can be defined as values that exceed the range of μ±kσ, where *k* is a constant, typically chosen as 2 or 3. If the covariance between certain nodes exceeds the range of μ±kσ, an anomaly is considered to have occurred, potentially indicating a network attack. For example, in reference [34], a residual and an evaluation function were established for fault detection. By applying the covariance linear learning algorithm, Albalawi et al. embedded feature selection to extract attributes highly correlated with IoT intrusions. They utilized the kernel-distributed Bayesian classifier to predict attacks accurately based on probability distribution values [35].

### 3.2. Data Mining Methods for Attack Detection

The primary steps in employing data mining methods for attack detection include association rule learning, clustering, classification, and regression. Data mining techniques facilitate effective feature sampling, the proactive prediction of visits, and a reduction in matching frequencies, thereby achieving proactive defense capabilities in attack detection.

Common approaches involve employing classification algorithms to devise classifiers for attack detection. This involves training a model to discern the feature distinctions between attack and normal samples within the dataset, thereby enabling the classification and evaluation of unknown samples. Kumar et al. examined the efficacy of support vector machines and decision trees in identifying DoS attacks, providing insightful comparisons between these techniques [36]. Kumari et al. developed a mathematical model that utilizes logistic regression to detect distributed DoS attacks, offering a robust approach to cybersecurity [37]. Further extending the discourse on machine learning’s role in security, Alsariera et al. introduced a specialized framework that employs two variants of decision tree algorithms to detect generic network intrusions [38]. In a similar vein, Reji et al. explored a hybrid machine learning model that combines the seagull optimization algorithm with an extreme learning machine classifier to enhance the detection and classification of cyber attacks [39]. The study in [40] proposed a mitigation strategy based on causal theory to enhance the robustness of ML-based network attack detection models in power systems against adversarial attacks, significantly reducing the need for adversarial samples and computing resources while maintaining transferability across various models. Kavousi-Fard et al. introduced a novel anomaly detection method for microgrids that uses prediction intervals and a modified optimization algorithm based on a symbiotic organism search to detect data integrity security in wireless sensor networks [41].

## 4. Secure State Estimation

In the context of a CPS under cyber attack, state estimation is crucial for maintaining accurate operation despite disruptions. Two prominent approaches to state estimation in such scenarios are the Kalman Filter and the Luenberger Observer. Each method applies distinct mathematical principles to estimate the system states efficiently.

### 4.1. State Estimation Based on the Kalman Filter

In the Kalman filter, state estimation involves a feedback control mechanism where the system’s state is estimated and then updated with measurement feedback. This process is structured around two main sets of equations: the time update equations, which predict the state and estimate error covariance for future states, and the measurement update equations, which refine these predictions by incorporating new, actual measurements to enhance the accuracy of the estimates. When noise is present in the system, ω(k) and ν(k) represent the process noise and measurement noise of the system, respectively, and are usually assumed to be mutually independent white noise with a normal distribution:

(12) p(ω)∼N(0,Q),p(ν)∼N(0,R).

The state estimation of the system using the Kalman filter is as follows.

Time updating equation:

(13) $$\begin{array}{c} \hat{x}{\left(k\right)}^{-}=A\hat{x}\left(k-1\right)+Bu\left(k\right), \end{array}$$

(14) $$\begin{array}{c} P^{-}\left(k\right)=AP\left(k-1\right)A^{T}+Q, \end{array}$$

where $\hat{x}{\left(k\right)}^{-}$ represents the a priori state estimation at *k* steps, $P^{-}\left(k\right)$ stands for the priori estimation error covariance, and *Q* is the process noise covariance.

Measurement update equation:

(15) $$\begin{array}{cc} \; & \;\;\;\;\;K\left(k\right)=P^{-}\left(k\right)C^{T}{\left(CP^{-}\left(k\right)C^{T}+R\right)}^{-1}, \end{array}$$

(16) $$\begin{array}{cc} \; & \;\;\;\;\hat{x}\left(k\right)=\hat{x}{\left(k\right)}^{-}+K\left(k\right)\left(y\left(k\right)-C\hat{x}{\left(k\right)}^{-}\right), \end{array}$$

(17) $$\begin{array}{cc} \; & P\left(k\right)=\left(I-K\left(k\right)C\right)P^{-}\left(k\right), \end{array}$$

where K(k) is the Kalman gain, *R* is the measurement noise covariance, x(k) denotes the posterior state estimate for a given measure in k steps, and P(k) is the posteriori estimation error covariance. The Kalman filter is a powerful tool for state estimation in systems with noisy measurements. It is particularly effective in linear dynamic systems but can be extended to nonlinear systems via its variants. In reference [42], a novel co-estimation method was introduced for the dynamic state estimation of CPS under sensor attacks, using Kalman filters and two-step Kalman filters’ preprocessing to enhance accuracy by considering the inter-correlations between CPS states and cyber-attacks. Combastel et al. introduced a distributed zonotopic and gaussian Kalman filter for robust state estimation in noisy, networked environments, utilizing symbolic zonotopes and Gaussian noise mergers for improved uncertainty propagation and dependency preservation [43]. A mathematical model complemented by a compensation strategy was introduced in [44] to address dimensionality reductions and communication delays in CPS. This model enhances information retention and establishes a recursive distributed Kalman fusion estimator. The study in [45] explored the design of FDI attacks in a distributed CPS, where each multi-hop network agent node enhances process estimation accuracy using Kalman-consensus filtering based on sensor data and inter-node communications.

### 4.2. State Estimation Based on the Luenberger Observer

The Luenberger observer is an essential tool in control theory, used for estimating the states of a dynamic system, especially when not all states are directly observable. Unlike the Kalman filter, which optimally estimates the states of a system with the known statistical properties of noise, the Luenberger observer does not require statistical knowledge about the noise and instead relies on full knowledge of the system’s dynamics. The Luenberger observer is generally in the following form:

(18) $$\begin{array}{c} \left\{\begin{array}{c} \hat{x}\left(k+1\right)=\left(A-LC\right)\hat{x}\left(k\right)+Bu\left(k\right)+Ly\left(k\right),\\ \hat{y}\left(k\right)=C\hat{x}\left(k\right), \end{array}\right. \end{array}$$

where $\hat{x}\left(k\right)$ is the estimated state and *L* is the observer gain.

In secure state estimation, the Luenberger observer’s ability to function effectively without precise noise statistics and its adaptability to various system disturbances make it a valuable choice for ensuring the reliability and security of estimations in dynamic environments. Lu et al. developed a switched Luenberger observer for secure state estimation in CPS under sparse sensor and disturbance attacks, introducing a new projection operator and sufficient conditions based on LMIs for an observer design that efficiently estimates states without iterative algorithms, even under switched target attacks [46]. Secure estimation strategies for distributed CPS-facing sparse actuator and sensor corruptions were developed in [47], employing consensus-based optimization and a secure observer design. The strategies introduce concepts such as sparsity repairability and include robust estimation methods like the distributed projected heavy-ball estimator and Luenberger-like observer. In [48], a Luenberger-type observer was used to estimate system dynamics and handle complex time series challenges in a neural network-controlled environment subject to DoS attacks and an adaptive event-triggered mechanism. Reference [49] introduced a novel resilient control system for load frequency control in smart power grids, incorporating a Luenberger observer and an artificial neural network enhanced by the extended Kalman filter for the rapid detection and mitigation of FDI attacks, ensuring system stability without the need for control reconfiguration.

## 5. Security Control

In CPS, security control under cyber attacks is critical due to the integrated nature of physical processes and computing devices. Cyber attacks, such as DoS or FDI, can disrupt operational stability and safety. Effective security controls are essential to mitigate risks, ensure system resilience, and maintain critical infrastructure integrity. There are several common methods of CPS security control, as follows.

### 5.1. Switching Control

Switching control involves dynamically changing the control law or system configuration in response to evolving conditions or system states. This can be formalized as follows:

(19) $$\begin{array}{c} \left\{\begin{array}{c} x\left(k+1\right)=A_{\sigma_{k}}x\left(k\right)+B_{\sigma_{k}}u\left(k\right),\\ z\left(k\right)=C_{\sigma_{k}}x\left(k\right), \end{array}\right. \end{array}$$

where $\sigma_{k}$ is the switching sequence determined by the attack signal, whereby the original system is modeled as a switching system containing the attacked subsystems and the normal operating subsystem. Switching control allows for the system to adapt to a range of operating conditions and attack scenarios by switching between different controllers or system modes. Switching control is pivotal when dynamically adjusting the control strategy in response to varying attack methods and targets, ensuring the continuous safe operation of a CPS. This approach leverages the flexibility of switching between different controllers or system states in real-time to respond to cyber threats effectively.

The study by Wang et al. [50] makes a significant contribution by developing a unified model to manage both zero-input and hold-input attacks. It innovatively reformulates a CPS as a switched system with time-varying delays, employing type-dependent average dwell time switching alongside multiple discontinuous Lyapunov functions. This method not only ensures global uniform exponential stability but also addresses the challenge of maintaining control under mixed DoS attacks, a scenario less explored in previous studies. In contrast, Kazemi et al. [30] focus on the precision of state estimation under attack conditions. They propose a method for finite-time secure dynamic state estimation that utilizes a network of local finite-time state estimators. Their approach is particularly notable for its robust detection algorithm, designed to exclude data from sensory nodes that are compromised by cyber-attacks. This method not only enhances the reliability of state estimation but also pinpoints the exact locations of cyber intrusions, providing analytical proofs of the feasibility and convergence of the estimation mechanisms, which fills a crucial gap in ensuring the accuracy of state information under cyber threats. Furthermore, the work by Wu et al. [51] expands on the model of a switching system by incorporating controllable pairs for dynamic updating. This adaptation significantly enhances the system’s defense against unpredictable attacks and aids in isolating compromised actuators, offering a new dimension to the adaptability of CPS under ongoing cyber threats. Lastly, Yuan et al. [52] introduce a hybrid-theoretical framework that integrates both physical control and cybersecurity subsystems. Their model dynamically switches between defense and attack states, thereby enhancing system resilience. This approach is particularly adept at handling DoS attacks and represents a holistic strategy that synergistically merges cybersecurity measures with physical system controls, highlighting a strategic integration gap in earlier models.

Each of these studies uniquely contributes to the field of switching control in a CPS, progressively closing critical research gaps. However, a common limitation of these approaches is the complexity of real-time implementation and the need for advanced computational resources. Future research could focus on simplifying these systems for practical applications and extending their robustness to newer forms of cyber threats.

### 5.2. Model Predictive Control

Model predictive control (MPC) uses a model of the system to predict future states over a horizon and optimizes control actions by solving a finite-time optimization problem at each control step:

(20) $$\begin{array}{c} \min_{U}\sum_{k=0}^{N-1}l\left(x\left(k|t\right),u\left(k|t\right)\right), \end{array}$$

subject to x(k|t)=f(x(k|t),u(k|t)), where *l* is the cost function, *f* is the system model, and *N* is the prediction horizon. MPC is a critical strategy in a CPS for proactively anticipating and adjusting to future system states to mitigate the effects of cyber-attacks. This approach is distinguished by its ability to integrate operational and security constraints directly into the control strategy, enhancing the system’s safety and resilience.

He et al. [53] advanced the field of MPC by introducing a resilient, self-triggered MPC strategy specifically designed for CPSs under FDI attacks. Their approach optimizes system stability and resource usage by dynamically adjusting the MPC update frequency. Notably, they enhance control data resilience through a novel input signal reconstruction mechanism, whose recursive feasibility and input-to-state stability have been rigorously proven. This study addresses the need for flexible control updates in response to dynamic threat environments, filling a gap in static MPC applications which do not account for variable attack timings and intensities. In another significant development, Geng et al. [54] developed an MPC strategy utilizing a type-2 Takagi-Sugeno fuzzy model to effectively counter multichannel jamming attacks. They introduced a strategic power allocation for jammers and an online fuzzy MPC algorithm that optimizes controller gains. This approach not only ensures system stability with guaranteed recursive feasibility but also introduces adaptability to the MPC framework in handling complex, multi-threat scenarios, a challenge that is often overlooked in traditional MPC designs. Sun et al. [55] presented a resilient MPC framework aimed at mitigating the impacts of DoS attacks on CPSs. Their framework underscores the necessity of adhering to specific MPC parameters and conditions tailored to the duration and nature of the attacks. Techniques such as the μ-step positively invariant set are employed to maintain system stability under these challenging conditions, highlighting the critical role of precise parameter tuning in ensuring effective defense against persistent network threats. Lastly, a groundbreaking MPC framework introduced by Zhang et al. [56] incorporates a disturbance observer and a memory module to address time-varying uncertainties in CPSs under DoS attacks. This framework is designed to maintain system stability and security by dynamically adjusting to the uncertainties caused by the attacks, illustrating a significant advancement in the field by integrating real-time data adjustment capabilities into the MPC strategy, a necessary evolution in the face of increasingly sophisticated cyber threats.

While each contribution significantly advances the application of MPC in cyber-threat environments, ongoing challenges include reducing the computational demands and enhancing the adaptability of these sophisticated models for their real-time and scalable use in practical settings.

### 5.3. Event-Triggered Control

In event-triggered control, decisions to update control inputs are made based on the occurrence of certain events rather than at fixed time intervals:

(21) $$\begin{array}{c} u(t)=u(kT)\;\mathrm{if}\;e(t)>\delta , \end{array}$$

where e(t) is the error signal, δ is a threshold, and *T* is the sampling period.

Event-triggered control significantly curtails network traffic and diminishes the likelihood of cyber attacks by initiating communications solely when necessary. This strategy proves invaluable for large-scale systems where persistent monitoring and constant updates are not feasible. In their groundbreaking work, Liu et al. [57] crafted a resilient control algorithm for CPSs under DoS attacks, integrating an event-triggered mechanism with sarsa learning to boost system stability and security. This innovative combination of learning algorithms sets it apart, allowing it to adaptively respond to evolving threat landscapes. Exploring further, Miao et al. [58] investigated an event-triggered sliding mode predictive control method tailored for remote motor systems vulnerable to DoS attacks. Their method prioritizes system stability with a sliding mode strategy adept at counteracting disruptions from sensor signals, which is crucial for preserving operational integrity during cyber assaults. Additionally, Ma et al. [59] devised a secure event-triggered control strategy for industrial CPSs challenged by resource limitations and deceptive attacks. They employed a neural network-based algorithm to adeptly manage interconnected data and utilized Nussbaum-type functions to address the unpredictability of attack signals, ensuring robust stability across all system loops, and thereby presenting a formidable defense against intricate attack vectors. Concluding with an advanced application, Zhao et al. [60] unveiled a dual security control system for nonlinear CPSs affected by actuator faults and DoS attacks. Their novel approach leverages edge computing to filter data and incorporates a discrete event trigger system that seamlessly manages faults and attacks through a Takagi–Sugeno fuzzy model, exemplifying a sophisticated amalgamation of cyber and physical defense mechanisms.

These studies collectively enhance the sophistication of event-triggered control in CPS by targeting the specific vulnerabilities posed by cyber threats. Despite these advancements, the quest to optimize these systems to ensure better scalability and quicker response times remains critical, especially for their application across diverse industrial scenarios.

### 5.4. Optimal Control

Optimal control is a mathematical framework designed to establish a control policy that optimizes the performance of a dynamic system over a given time period. This involves finding a control function that will minimize or maximize a certain “cost” or “performance” index when applied to a system whose behavior can be described by differential equations. A standard optimal control problem can be formulated as follows:

(22) $$\begin{array}{c} J=\int_{t_{0}}^{t_{f}}L\left(x\left(t\right),u\left(t\right),t\right)dt, \end{array}$$

where *L* is the cost function, $t_{0}$ is the initial time, and $t_{f}$ is the final time.

Optimal control provides the framework used to design proactive strategies that effectively mitigate potential cyber threats. By embedding security measures directly within the control mechanisms, a CPS can sustain operational integrity across a range of attack scenarios. In pioneering work, Fei et al. [61] innovated a model-free Q-Learning algorithm tailored for optimal control in CPSs facing DoS and FDI attacks, utilizing a non-cooperative Stackelberg game framework. This approach successfully derives an optimal control policy via a game algebraic Riccati equation (GARE), establishing conditions that ensure a solution to GARE and leveraging Q-Learning to ascertain this solution independently of system dynamics or state knowledge. Further advancing the field, the same researchers introduced data-driven methodologies and zero-sum game theory to formulate advanced control strategies for a CPS under FDI threats [62]. Their development of dual Q-learning algorithms facilitates a model-free control design, obviating the need for traditional system models and state vectors and thus broadening the practical application of these controls. Additionally, Wu et al. [63] delved into a zero-sum game-based optimal control strategy specifically for CPSs compromised by actuator FDI attacks. This strategy is crafted using a dynamic programming approach within an infinite-horizon quadratic cost framework, yielding optimal defense and attack policies that enhance the robustness of the CPS against sophisticated cyber manipulations.

These contributions signify crucial steps forward in the application of optimal control to enhance CPSs’ security. Nonetheless, the continuous evolution of cyber threats necessitates ongoing research to refine these strategies, ensuring they remain effective against increasingly sophisticated attacks and are adaptable to new technological landscapes.

## 6. Future Work

In the domain of CPS security control, several unresolved issues persist, posing challenges and driving ongoing research. These challenges include the following: As CPSs become larger and more complex, ensuring security at scale without a significant loss in performance or a prohibitive increase in computational resources remains a challenge. Many CPS security controls are still not fully equipped to handle advanced and evolving cyber threats, such as polymorphic malware, zero-day attacks, and sophisticated persistent threats that can adapt and evade current detection methods. While AI and machine learning offer promising avenues for improving security detection and response, integrating these technologies into CPSs in a way that maintains robustness and does not introduce new vulnerabilities is a complex challenge. CPSs often require real-time or near-real-time processing for control and operation. Implementing security measures that can operate effectively at such speeds without causing system delays is challenging. CPSs involve both physical and cyber components, and security solutions must effectively integrate protections for both aspects. Many current strategies are siloed, focusing on either physical or cyber threats, but not both concurrently.

## 7. Conclusions

In reviewing the security control for CPSs under cyber-attacks, we conducted a thorough examination of prevalent cyber threats such as DoS, FDI, and replay attacks. Each of these attacks presents unique challenges in terms of detectability, the necessity for system information, and potential destructiveness. DoS attacks are relatively easy to detect due to their disruptions to service availability but do not necessarily require detailed system information to be executed. FDI attacks demand in-depth system knowledge to manipulate data effectively and are more challenging to detect because they blend in by mimicking legitimate data. Replay attacks involve capturing and retransmitting valid data; they require access to system data and are moderately detectable since they do not alter the original data.

The article also details classic detection methods, describing their characteristics and the advantages and disadvantages inherent to each. Probability-based statistical methods, for example, are effective in detecting anomalies by comparing expected behavior against observed behavior, but they may suffer from high false-positive rates if the model of normal behavior is not accurately defined. Data mining methods can robustly uncover hidden patterns and anomalies in large datasets, though they demand extensive computational resources and can be complex to implement.

When focusing specifically on security control strategies for CPSs, several approaches offer varied benefits and challenges. Event-triggered control reduces network traffic and conserves resources by activating communications only when significant events occur, making it suitable for large-scale CPSs where constant monitoring is infeasible. However, this method might overlook subtle, gradual anomalies that do not trigger immediate thresholds. Switching control provides the flexibility to adapt control strategies dynamically in response to identified threats, enhancing the system’s resilience. This adaptability is crucial in environments where attack modes can change rapidly. However, managing the complexity of multiple control schemes can be challenging.

Predictive control uses system modeling to predict future states and adjust controls proactively, which is particularly effective in thwarting attacks before they cause harm. This anticipatory action is ideal for high-stakes CPS environments, like power grids or transportation systems. However, its effectiveness heavily relies on the accuracy of the predictive models that are used. Finally, optimal control seeks to optimize system performance under constraints, which is vital for maintaining CPSs’ efficiency and effectiveness during cyber threats. It systematically addresses the trade-offs between security measures and system performance but requires complex calculations, which can be a barrier to real-time applications.

In conclusion, enhancing CPS security requires a nuanced understanding of various cyber threats and the tailored application of detection and control methods. A robust security framework for CPSs should integrate multiple strategies to address the specific vulnerabilities and threats associated with different cyber attack types. As we continue to advance our methods and technologies in response to the evolving threat landscape, focusing on adaptive, model-based, and resource-efficient strategies will be key to safeguarding the essential services and infrastructures dependent on secure and resilient CPSs.

## Data Availability

The datasets generated and analyzed during the current study are not publicly available but are available from the corresponding author on reasonable request.
